# Aortic dissection precipitated by superwarfarin (brodifacoum) poisoning: a case report and pathophysiological review

**DOI:** 10.3389/fsurg.2026.1756737

**Published:** 2026-08-19

**Authors:** Yuxin Xiao, Yutao Wang, Kuiquan Song, Yan Sun

**Affiliations:** 1 The First Clinical Medical College, Shandong University of Traditional Chinese Medicine, Jinan, China; 2 Department of Peripheral Vascular Disease, Guang'anmen Hospital Jinan Hospital, China Academy of Chinese Medical Sciences (Jinan Municipal Hospital of Traditional Chinese Medicine), Jinan, China; 3 Department of Vascular Surgery, Shandong Provincial Hospital, Jinan, China

**Keywords:** aortic dissection, brodifacoum, case report, coagulopathy, superwarfarin

## Abstract

**Background:**

Poisoning by rodenticides, particularly anticoagulant agents, has been well-documented. Brodifacoum, a second-generation “superwarfarin”, is a long-acting anticoagulant rodenticide (LAAR) characterized by an extended half-life. Its toxicity primarily manifests as excessive or abnormal bleeding from the skin, mucous membranes, gastrointestinal tract, and urinary tract. However, aortic dissection (AD)—a rare and catastrophic medical event—has seldom been reported as a complication of such poisoning. We present a case of extensive aortic dissection triggered by Brodifacoum poisoning. This case aims to elucidate the potential pathophysiological link between superwarfarin intoxication and the development of aortic dissection.

**Case presentation:**

A 69-year-old man was admitted to the hospital with a 9-day history of oral bleeding and an 8-day history of pain in the lower back, abdomen, and chest. Initial examinations at another facility were inconclusive. Upon transfer to our hospital, computed tomography angiography (CTA) revealed an extensive aortic dissection extending from the distal abdominal aorta to the bilateral iliac arteries. Key coagulation tests on admission indicated severe coagulopathy: both prothrombin time (PT) and the international normalized ratio (INR) were undetectable, and the activated partial thromboplastin time (APTT) was markedly prolonged to 160.1 s. Concurrent hematological tests revealed anemia (hemoglobin 102 g/L). Toxicological screening confirmed the presence of Brodifacoum in his blood. The patient's condition was stabilized through aggressive management, including infusion of fresh frozen plasma (FFP), high-dose intravenous vitamin K, and strict blood pressure control. His coagulation parameters gradually normalized over two weeks.

**Conclusion:**

This case highlights a life-threatening complication of superwarfarin poisoning—aortic dissection. It underscores the critical importance of considering toxicological etiologies in patients with unexplained coagulopathy. Early reversal of the coagulation deficit and rigorous blood pressure control are paramount in preventing the progression of dissection.

## 1 Introduction

Aortic dissection (AD) is a catastrophic vascular emergency associated with high mortality. Its classic presentation is the abrupt onset of severe, tearing pain (1, 2).However, atypical presentations often lead to diagnostic delays.

The vitamin K antagonist family originates from 1933 reported moldy sweet clover-induced bovine hemorrhagic disorder, which is mediated by dicoumarol. Warfarin, synthetized in 1948 and initially marketed as rodenticide, was later approved for human anticoagulation in 1954, sharing the same vitamin K epoxide reductase inhibitory mechanism with brodifacoum. A fatal case highlighted the hazard of unsupervised warfarin therapy: a mechanical mitral valve patient self-medicated warfarin plus aspirin for 2 years without INR monitoring, resulting in INR >6.6 and lethal intracerebral hemorrhage despite timely hemostatic rescue; the patient eventually died of ICU-acquired sepsis (3). This uncontrolled iatrogenic anticoagulation resembles brodifacoum poisoning, yet brodifacoum's far longer half-life causes refractory coagulopathy and rare aortic dissection as seen in our case.

Brodifacoum is long-acting anticoagulant rodenticides (LAARs). These agents exert their toxicity by inhibiting vitamin K epoxide reductase, which leads to a severe deficiency of vitamin K-dependent clotting factors (II, VII, IX, X). Consequently, patients with superwarfarin poisoning typically present with signs and symptoms of bleeding and hemorrhagic diathesis (4, 5).This report describes a case of extensive aortic dissection in a patient whose initial symptoms were oral bleeding and multi-site pain. The condition was ultimately diagnosed as severe acquired coagulopathy secondary to superwarfarin (Brodifacoum) poisoning. This case underscores an important diagnostic consideration in clinical practice.

## 2 Case presentation

### 2.1 Initial presentation and diagnostic process

A 69-year-old man was admitted to our hospital with a 9-day history of oral bleeding and an 8-day history of pain involving the lower back, abdomen, and chest. His medical history was unremarkable for hypertension or other underlying conditions, and he had no history of anticoagulant medication use. Six days prior to admission, computed tomography (CT) and ultrasonography performed at another hospital revealed no significant abnormalities in the upper abdomen on CT. However, edema was noted around the origins of the bilateral common iliac arteries, and contrast-enhanced scanning was recommended for further evaluation. Iliac artery ultrasonography demonstrated bilateral iliac atherosclerosis with plaque formation. These initial findings were inconclusive for a definitive diagnosis. His pain symptoms were partially and transiently relieved with analgesic treatment. Three days before admission, he developed pain in his right calf and both upper limbs. Vascular ultrasonography performed on admission revealed a aortic dissection extending from the distal abdominal aorta to the bilateral iliac arteries, accompanied by an intramural hematoma.

### 2.2 Discovery and final diagnosis of coagulation dysfunction

**Figure 1:**
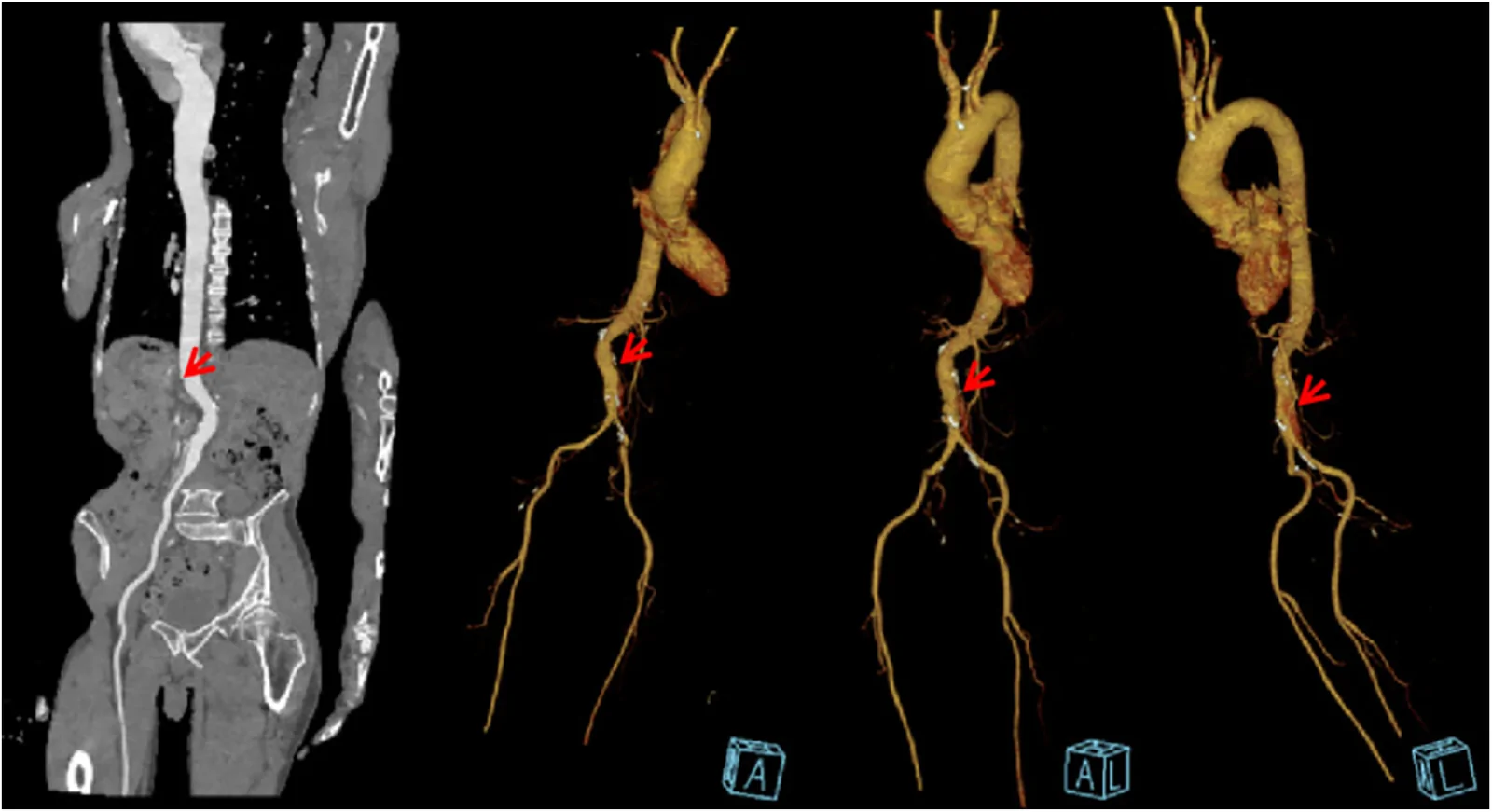
Initial admission CTA left (MPR): An intimal tear (arrow) at the abdominal aorta inferior to L3, with contrast flowing from true to false lumen. Right (3D VR):Views from theanterior, anterior-right oblique, and anterior-left oblique projections show the tear (arrows) on the left anterior wall of the distal aorta. The dissection extends distally to bilateral iliac arteries with false lumen expansion.

**Figure 2:**
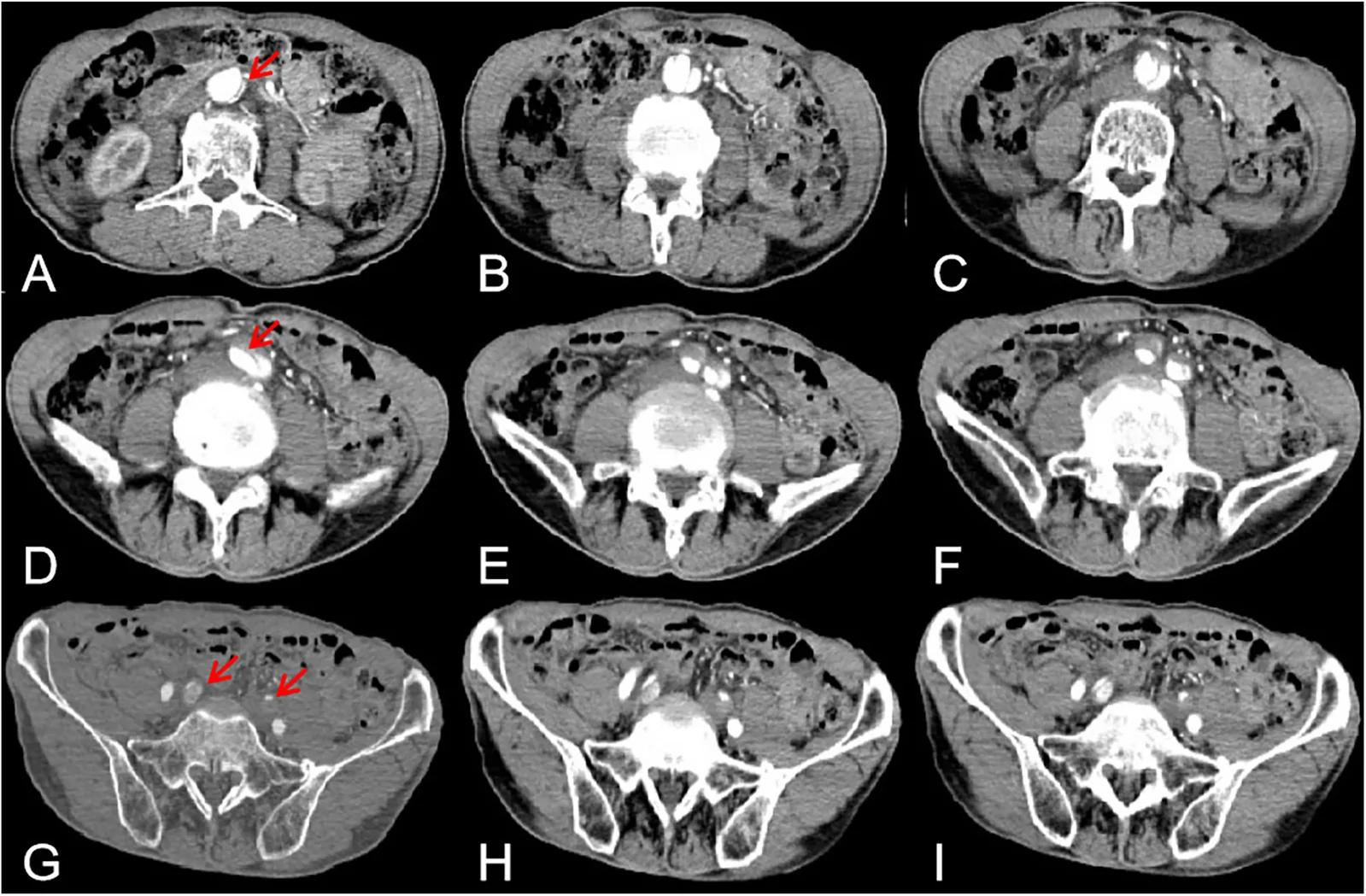
Initial admission CTA **(A–C)** distal abdominal aorta level: demonstrates the initiation of the dissection flap (arrow) and associated intramural hematoma. **(D–F)** Aortoiliac bifurcation level: Shows the extension of the dissection (arrow) into the common iliac arteries. **(G–I)** Iliac artery level: Reveals the dissection extending into the left internal iliac artery and right internal iliac artery (arrows).

Physical examination on the day of admission revealed a blood pressure of 146/96 mmHg, active oral bleeding, and a large ecchymosis on the right upper limb. Laboratory investigations indicated severe coagulopathy: both prothrombin time (PT) and the international normalized ratio (INR) were unmeasurably high, and the activated partial thromboplastin time (APTT) was markedly prolonged to 160.1 s. He also had normocytic anemia (hemoglobin 102 g/L). Further laboratory investigations revealed normal liver function (alanine aminotransferase 9.20 U/L and aspartate aminotransferase 14.90 U/L) and renal function (creatinine 55.0 μmol/L), effectively ruling out primary hepatic or renal insufficiency as the cause of the coagulopathy. Aortic computed tomography angiography (CTA) confirmed an extensive aortic dissection involving the distal abdominal aorta down to the bilateral iliac arteries (Figures 1, 2).

The patient's family could not provide a definitive history of rodenticide exposure. The patient himself, upon recovery, recalled possibly ingesting contaminated food in the days preceding symptom onset, though the exact source was unclear. Given the profound coagulopathy in the absence of anticoagulant use, normal hepatorenal function, and this unclear exposure history, a toxicological screen using gas chromatography/mass spectrometry (GC/MS) was performed on serum. The test returned a high serum concentration of Brodifacoum at 77.86 ng/mL.The final diagnoses were: 1) extensive aortic dissection, and 2) severe acquired coagulopathy due to superwarfarin (Brodifacoum) poisoning.

### 2.3 Treatment and follow-up

Upon diagnosis of superwarfarin-induced coagulopathy complicating aortic dissection, a dual-strategy therapeutic regimen was immediately implemented. The primary objectives were the aggressive reversal of coagulopathy and the strict control of hemodynamics to prevent dissection progression.

First, to urgently correct the life-threatening coagulopathy, a combination of fresh frozen plasma (FFP) and high-dose vitamin K1 was initiated. The patient received an initial bolus transfusion of 300 mL of FFP upon admission, followed by daily transfusions of 400 mL for the next five consecutive days to maintain adequate levels of functional clotting factors. Concurrently, high-dose intravenous vitamin K1 was administered at 10 mg every 12 h. This intensive dosing regimen was selected based on the severe degree of poisoning (serum brodifacoum level: 77.86 ng/mL) and aligns with recommendations for managing significant superwarfarin intoxication, where sustained vitamin K repletion is critical. As the patient's coagulation parameters demonstrated consistent improvement (see Table 1 for detailed trends), the vitamin K1 regimen was successfully tapered to 10 mg once daily.

**Table 1:** Temporal Profile of Coagulation Parameters, Hematological Values, and Therapeutic Management.

DOH	PT	INR	APTT	Hb	PLT	VK	FFP
1	Unmeasurable	Unmeasurable	160.1	102	545	10 mg;q12h	300mL
2 (am)	92.4	9.03	77.4	88	492	10 mg;q12h	400mL
2 (pm)	37.9	3.51	47.6	82	463		
3	40.5	3.76	43.3	82	479	10 mg;q12h	400mL
4	34.8	3.24	34.4	91	551	10 mg;q12h	400mL
5	33.5	3.11	32.2	88	556	10 mg;q24h	400mL
6	28.4	2.58	30.2	88	581	10 mg;q24h	400mL
7	28.1	2.55	26.9	92	587	10 mg;q24h	
8	30.0	2.76	29.8	88	551	10 mg;q24h	
12	21.6	1.93	29.0	92	424		
17	18.5	1.64	27.5	97	336		

Normal range: PT:10-14;INR: 0.7-1.3;APTT: 22-35;Hb:110-160;PLT:100-400.

DOH: Day of hospitalization, with the day of admission designated as DOH 1.

Second, stringent hemodynamic control was instituted to minimize shear stress on the dissected aortic wall.​ Targets were set at a systolic blood pressure of 100–120 mmHg and a heart rate of 60–80 beats per minute, consistent with guideline-directed management of aortic dissection. This was achieved with oral metoprolol sustained-release (47.5 mg once daily) to reduce heart rate and contractility, and oral felodipine sustained-release (5 mg once daily) for vasodilation. This combination of a cardioselective beta-blocker and a vasodilator was chosen for its synergistic effect in reducing aortic wall stress. The β1-selectivity of metoprolol was preferred to avoid the potential vasodilatory effects of non-selective beta-blockers, which could theoretically exacerbate bleeding tendency.

**Figure 3:**
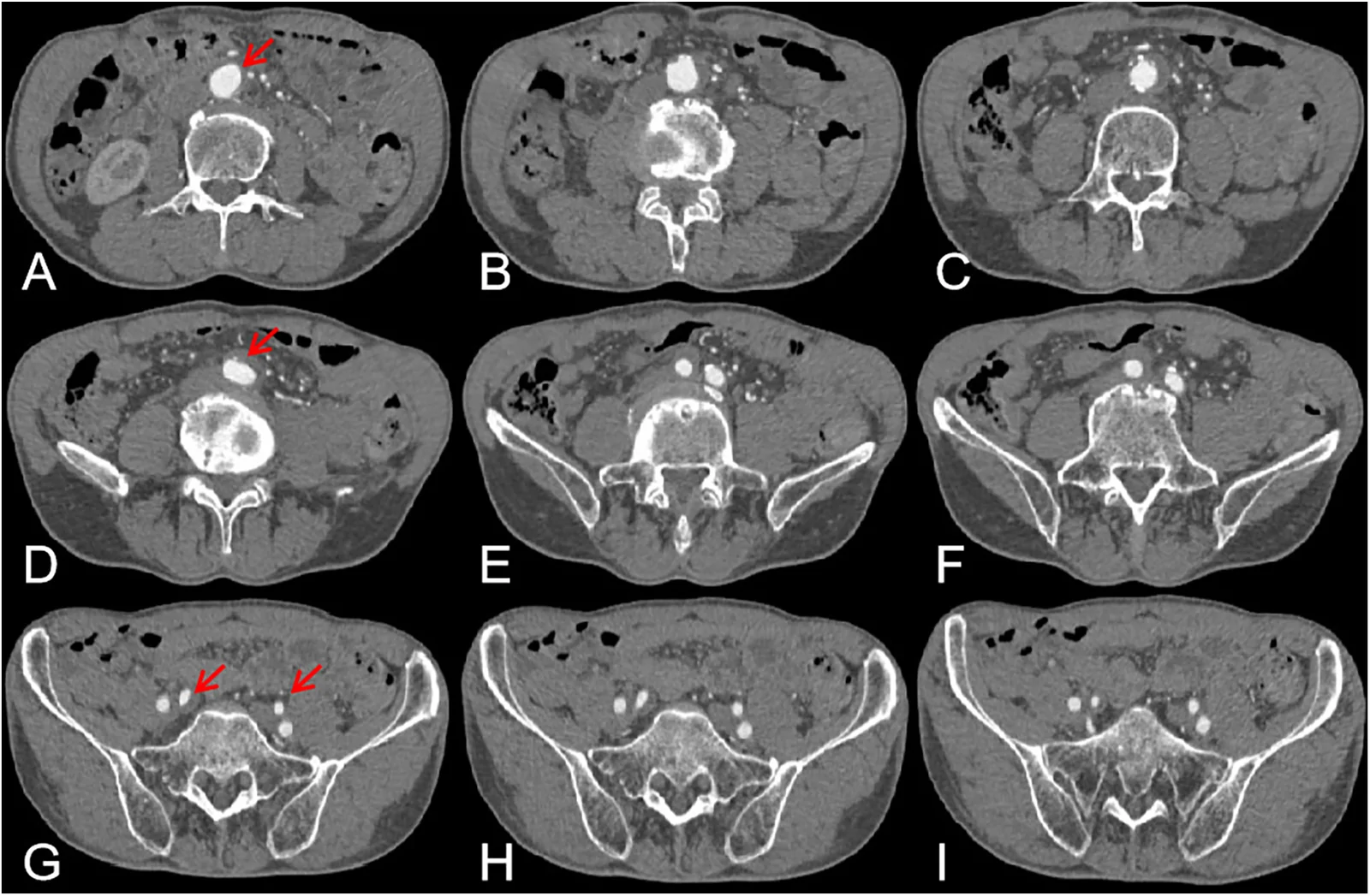
Follow-up CTA after initial treatment **(A–C)** distal abdominal aorta level: the dissection flap (arrow) persists with stable intramural hematoma. **(D–F)** Aortoiliac bifurcation level: The dissection (arrow) remains unchanged, showing no interval progression. **(G–I)** Iliac artery level: The dissection in the iliac arteries (arrows) is stable, with reduced hematoma density indicating early organization.

**Figure 4:**
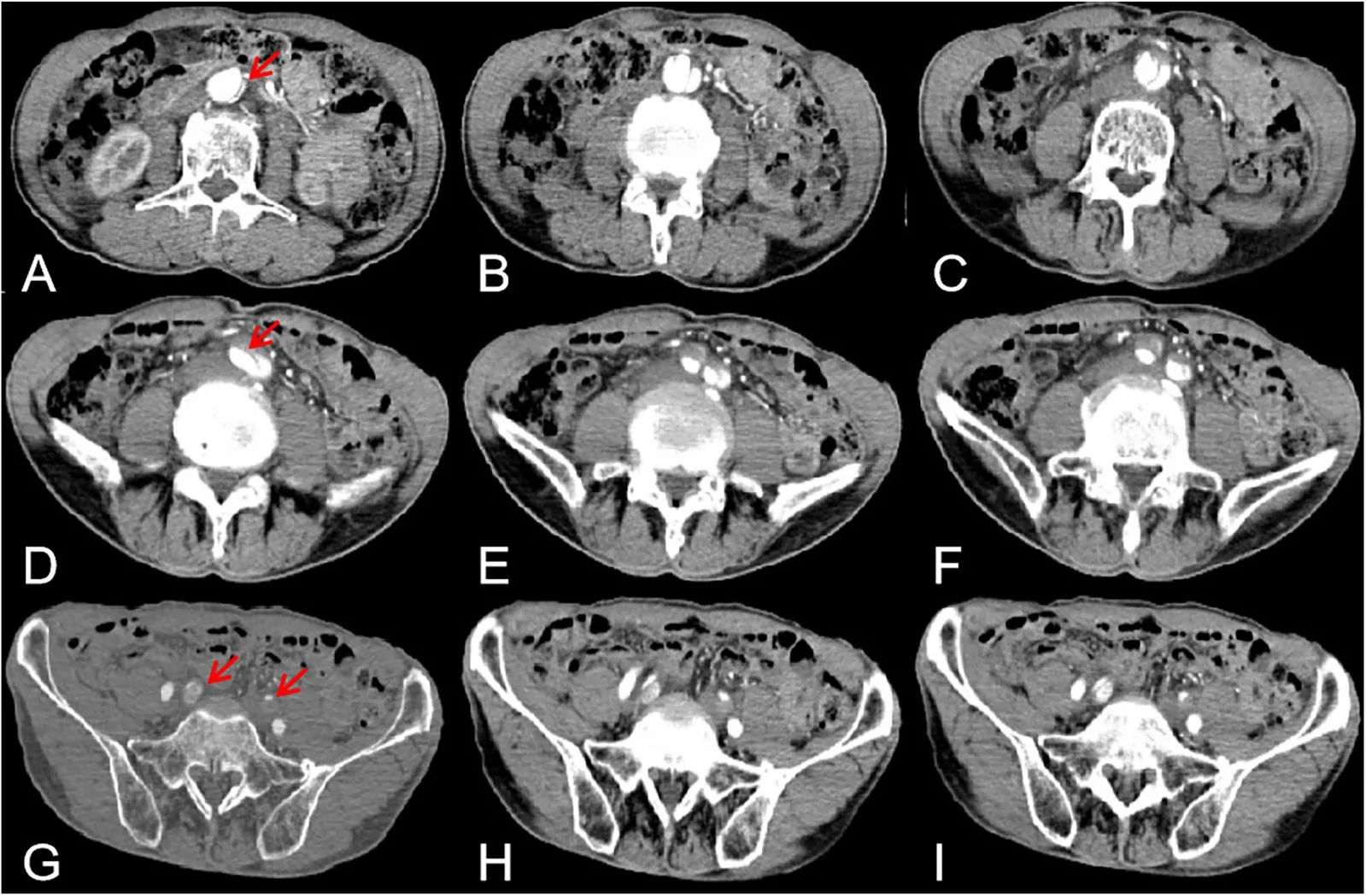
One–month posT,discharge surveillance CTA **(A–C)** distal abdominal aorta level: stable appearance of the dissection flap (arrow) with organized intramural hematoma. **(D–F)** Aortoiliac bifurcation level: The dissection (arrow) remains stable with no significant interval change. **(G–I)** Iliac artery level: The iliac artery dissection (arrows) shows complete stability with further hematoma resolution.

Finally, the patient's clinical course and follow-up are summarized. He responded favorably to the combined regimen, with coagulation parameters (PT, INR, APTT) showing progressive normalization over two weeks (Table 1). Serial imaging monitored the aortic pathology. A follow-up CTA approximately two weeks after admission showed that the dissection from the distal abdominal aorta to the bilateral iliac arteries persisted, but the associated intramural hematoma had stabilized without progression (Figure 3). An outpatient surveillance CTA one month after discharge revealed further positive evolution: the dissection flap and intramural hematoma remained completely stable at all levels, with evidence of hematoma resolution (Figure 4). The patient was discharged with a plan for long-term outpatient follow-up to monitor both aortic and coagulation stability.

## 3 Discussion

This case illustrates a rare and life-threatening complication of superwarfarin poisoning: aortic dissection. The distinctiveness of this case lies in its presentation with occult coagulopathy and migratory pain, coupled with non-diagnostic initial imaging findings, which collectively led to a delayed diagnosis. The profound acquired coagulopathy was subsequently confirmed through toxicological screening to be due to Brodifacoum poisoning, a type of superwarfarin. Aortic dissection resulting from superwarfarin intoxication is exceedingly rare, and its underlying pathophysiology is particularly complex.

### 3.1 Pathophysiological mechanism

We propose that the development of aortic dissection in this case followed a multi-step and synergistically amplified pathophysiological cascade, initiated by severe superwarfarin-induced coagulopathy. This postulation is supported by a clear temporal relationship, wherein markers of coagulopathy preceded the onset of pain.

Brodifacoum inhibits vitamin K epoxide reductase and thus the recycling of the inactive vitamin K epoxide back to the active reduced form of vitamin K, thereby disrupting the activation of vitamin K-dependent clotting factors (II, VII, IX, X) and leading to severe coagulopathy (6, 7). Beyond the manifest superficial hemorrhagic manifestations such as oral bleeding and cutaneous ecchymosis, a more critical consequence was the induction of spontaneous hemorrhage within the aortic wall itself (8). The patient's pre-existing aortic atherosclerosis likely served as a structural vulnerable site within the vascular system (9).On one hand, the vasa vasorum of the arterial wall become prone to rupture in the anticoagulated state. On the other hand, unstable atherosclerotic plaques are rich in friable neovessels (10, 11), which represent the most susceptible sites for bleeding in the setting of systemic anticoagulation. Hemorrhage from these micro-ruptures can accumulate within the medial layer of the aortic wall, forming an initial intramural hematoma (12).

Subsequently, a hyperdynamic circulatory state, induced by anemia, acted synergistically with the patient's underlying hypertension to amplify mechanical stress on the aortic wall. Persistent bleeding led to moderate-to-severe anemia, with hemoglobin decreasing from 102 g/L to 88 g/L, which in turn triggered a compensatory hyperdynamic state (13).This state is characterized by an increased cardiac output, which elevates aortic flow velocity and volume, thereby significantly augmenting wall shear stress (14).The patient's underlying hypertension further exacerbated this effect by increasing aortic wall stress and, in synergy with the hyperdynamic circulation, pathologically elevating hemodynamic shear forces through enhanced blood flow impact on the vessel wall (15).

Ultimately, the markedly elevated mechanical stress culminated in an intimal tear. The abnormally high hemodynamic shear stress persistently acted upon the aortic intima, which was already compromised and under elevated tension due to the intramural hematoma. Concurrently, the severe pain from the dissection activated the sympathetic nervous system, causing tachycardia and vasoconstriction (16). This “second hit” ultimately led to a focal intimal tear (17, 18).Once an intimal tear was established, blood, driven by high pressure, entered the medial layer and propagated longitudinally, ultimately resulting in the extensive dissection observed in this case, which extended from the abdominal aorta to both iliac arteries. Although a direct causal link between superwarfarin and aortic dissection is exceedingly rare, the mechanism of spontaneous intramural hematoma formation due to over-anticoagulation is well-documented (19), providing a plausible explanation for the causal inference in this case.

### 3.2 Literature context: anticoagulant-associated aortic dissection and the uniqueness of the present case

Although the association between anticoagulation and aortic dissection is not incorporated into current guideline recommendations, it has repeatedly surfaced in clinical practice as a context-dependent dilemma. Existing reports almost exclusively describe this phenomenon in the setting of therapeutic anticoagulation, where the risk–benefit calculus differs fundamentally from the toxicant-induced coagulopathy presented here.

Case reports and series have documented the occurrence or progression of aortic dissection in patients receiving anticoagulants for standard medical indications, such as atrial fibrillation or left ventricular thrombus (19–21). A seminal report by Blunt and Impallomeni described an 80-year-old woman on long-term warfarin who developed an acute type B thoracic aortic dissection coincident with an elevated INR of 4.8, highlighting intramural hemorrhage as a plausible mechanism (19).This scenario underscores a critical clinical dilemma: balancing the imperative for anticoagulation against the potential to exacerbate aortic pathology. Notably, some evidence suggests that with meticulous monitoring, anticoagulation and aortic stability may coexist. For instance, Bocchino et al. reported a case of type B dissection with a left ventricular thrombus that remained stable under combined warfarin and antiplatelet therapy (20). Similarly, Cañadas et al. observed the absorption of aortic intramural hematoma in patients maintained on anticoagulation (22). This body of literature is complemented by studies of novel agents; Yildirim et al. reported the first case of a patient with chronic type B dissection safely treated with dabigatran for atrial fibrillation over a six-month follow-up (23). Conversely, the study by Davis et al. serves as a critical warning, demonstrating that inadvertent anticoagulation in emergency department patients later diagnosed with aortic dissection was associated with significantly worse hemorrhagic outcomes (24). Collectively, these reports delineate a clinical spectrum centered on the managed risk of controlled, therapeutic anticoagulation in patients with known or subsequent aortic disease.

Beyond vitamin K antagonists, Xa inhibitor DOACs carry elevated bleeding risks when co-administered with CYP3A4/P-gp inhibitors such as diltiazem. Diltiazem raises apixaban AUC by 40% and Cmax by 31%; concurrent low-dose aspirin further synergizes anti-hemostatic effects and induces fatal hemorrhage. One reported 69-year-old polymorbid cardiovascular patient took unadjusted apixaban, diltiazem and aspirin, presenting with severe upper gastrointestinal bleeding and hemorrhagic shock (Hb 7.4 g/dL). Liver and renal function were normal, confirming triple-drug interaction as the sole trigger; he died from ventilator-associated sepsis after hemorrhage control (25).This educational case strongly highlights the critical clinical significance of DOAC-related drug-drug interactions. Regrettably, the fatal adverse outcome in this patient stemmed from inadequate medication risk assessment and neglect of pharmacokinetic interplay among multiple agents by clinical staff. Without timely dosage reduction or alternative antihypertensive replacement, the synergistic antihemostatic effect accumulated progressively and ultimately led to irreversible life-threatening bleeding complications, serving as a vital clinical warning for all frontline clinicians managing patients on oral anticoagulants. Clinically distinguishable differences separate DOAC-interaction bleeding from our brodifacoum-induced aortic dissection. DOAC-related hemorrhage stems from titrated therapeutic anticoagulation amplified by prescription drug interactions, with only mild elevation of anti-Xa activity and intact vitamin K-dependent clotting factors, mainly manifesting as mucosal gastrointestinal bleeding. In contrast, brodifacoum fully abolishes functional II/VII/IX/X factors, triggering aortic vasa vasorum rupture, intramural hematoma and extensive dissection—a severe vascular complication unreported in DOAC-related bleeding. Clinicians should differentiate these two anticoagulation-mediated fatal events for targeted management.

In stark contrast to the above scenarios, our case introduces a fundamentally different etiology and high-risk paradigm: acute aortic dissection precipitated by severe, uncontrolled coagulopathy from brodifacoum poisoning. Table 2 summarizes these key distinctions, which are elaborated below. Unlike the iatrogenic anticoagulation covered above, superwarfarin poisoning constitutes an independent clinical entity with three key distinguishing features. First, regarding etiology and intent, the anticoagulant state stems from accidental toxic ingestion instead of intentional medical treatment, placing it in the category of public health poisoning rather than clinical antithrombotic management. Second, in terms of hemostatic disturbance severity, brodifacoum leads to complete, unmanageable failure of coagulation function far beyond any acceptable therapeutic INR window, which differs greatly from the adjustable, targeted pharmacological effect of prescribed anticoagulants. Third, from a diagnostic perspective, the coexistence of catastrophic aortic syndrome and unexplained severe coagulopathy requires reverse clinical reasoning: clinicians must first identify the toxic origin of bleeding diathesis to interpret the aortic lesion, a workflow completely distinct from patients receiving planned anticoagulant therapy.

**Table 2:** Contrasting features of reported anticoagulant-associated aortic dissection cases.

Feature	Therapeutic Anticoagulation-Associated Cases	Present Case (Toxicant-Induced)
Primary Context	Medical therapy for conditions (e.g., AF, VTE)	Accidental superwarfarin (brodifacoum) poisoning
Anticoagulant Goal	Controlled, dose-adjusted therapy	Uncontrolled, toxic effect
Typical Coagulation Status	Therapeutic INR range (e.g., 2.0–3.5)	Profoundly supratherapeutic (INR >10)
Core Clinical Dilemma	Balancing thrombosis prevention vs. dissection/bleeding risk	Diagnosing the cause of coagulopathy to guide reversal and aortic management
Representative Examples	Warfarin use in AF (19); DOAC use in chronic dissection. (23); anticoagulation decision-making in chronic dissection (21); Fatal triple-drug interaction of apixaban, diltiazem and aspirin (25)	First detailed report of extensive abdominal aortic dissection precipitated by superwarfarin poisoning
Public Health Implication	Drug safety monitoring within medical practice;Clinical alertness for life-threatening DOAC pharmacokinetic interactions (25)	Highlights risks of potent, long-acting anticoagulant rodenticides

While the relationship between therapeutic anticoagulation and aortic dissection is complex and context-dependent, the present case establishes severe toxicant-induced coagulopathy as a novel and under-recognized precipitating factor for acute aortic dissection. It expands the differential diagnosis for “aortic syndrome with unexplained coagulopathy” beyond medical causes to include toxicological emergencies. This underscores the imperative for clinicians to consider superwarfarin poisoning in such presentations and reinforces the significant public health risk posed by these accessible, long-acting anticoagulants.

### 3.3 Therapeutic strategy and rationale

The management of this case was guided by two fundamental principles: aggressive reversal of the coagulopathy and strict control of hemodynamic parameters. The overarching goal was to eliminate the driving forces for dissection progression.

For the management of coagulopathy, a replacement therapy regimen consisting of FFP and high-dose intravenous vitamin K was administered. FFP provides rapid replenishment of immediately required coagulation factors, aiming to emergently control active bleeding and stabilize circulation. Concurrent high-dose intravenous vitamin K (10 mg every 12 h) was administered to bypass the enzyme pathway inhibited by Brodifacoum and to restart the synthesis of endogenous coagulation factors. This combination regimen represents the standard-of-care for managing life-threatening hemorrhage caused by superwarfarin poisoning (4, 9).As demonstrated in Table 1, this combination therapy acted rapidly, with significant improvements in PT, INR, and APTT observed within 24 h, and these parameters trended toward normalcy within two weeks. This confirms the critical importance of sustained and adequate vitamin K therapy to counteract the prolonged effect of superwarfarins (4).It is noteworthy that four-factor prothrombin complex concentrate (PCC) is considered an effective alternative to FFP for rapid reversal, offering advantages in volume load and precise factor content. In this case, the treatment team's decision to utilize FFP was based on specific institutional protocols and immediate drug availability, a pragmatic choice given that FFP remains a guideline-recommended agent for anticoagulation reversal alongside alternatives like PCC (26).

Regarding hemodynamic management, a strategy of tight blood pressure and heart rate control (systolic blood pressure 100–120mmHg, heart rate 60–80 beats per minute) was implemented to minimize hemodynamic forces that promote dissection propagation (6).The selected drug combination—a long-acting calcium channel blocker (felodipine) and a selective β1-blocker (metoprolol)—has a well-established synergistic effect. Felodipine lowers blood pressure steadily via peripheral vasodilation, whereas metoprolol effectively reduces the velocity of left ventricular ejection and its impact on the aortic wall by decreasing heart rate and myocardial contractility. This pharmacological combination is crucial for reducing shear stress on the aortic wall (15).It is noteworthy that, unlike non-selective beta-blockers with *α*1-blocking activity, metoprolol is a highly selective β1-receptor blocker. This selectivity is advantageous as it does not adversely affect platelet aggregation or vasoconstriction, and is thus not expected to exacerbate bleeding tendency (27).This property rendered it particularly suitable for the present case complicated by severe coagulopathy.

Given that the patient's coagulopathy was corrected with the aforementioned medical therapy, and the dissection and intramural hematoma had stabilized showing signs of resolution, a decision was made to continue conservative management rather than pursue surgical or endovascular intervention. This decision was based on the assessment that the root cause (coagulopathy) had been effectively controlled and the dissection itself had entered a stable phase.

### 3.4 Epidemiological context and diagnostic consideration

The clinical presentation of severe coagulopathy without an apparent cause necessitates a broad differential diagnosis, with superwarfarin poisoning being a critical consideration. This case aligns with an emerging public health concern regarding unintentional exposure to long-acting anticoagulant rodenticides， which are widely used in global agricultural and urban rodent control due to their high efficacy. In China, the use of these compounds is regulated under the Pesticide Administration Regulation. Specifically, agents like brodifacoum are listed in the *Catalog of Restricted-Use Pesticides* as an official catalog published by the Ministry of Agriculture and Rural Affairs, mandating strict controls on their production, distribution, and application (28).This “restricted-use” policy, which mandates licensing for production, specialized distribution, and documented application, aims to mitigate public health risks while acknowledging their role in professional pest management. However, challenges in consistent policy implementation at the local level, including issues of illicit circulation and improper disposal, persist as potential sources of unintentional exposure. Notably, exposure routes extend beyond direct contact with rodenticides. There are internationally documented outbreaks of coagulopathy linked to synthetic cannabinoids adulterated with LAARs, confirming that oral ingestion via a contaminated vehicle constitutes a potent exposure pathway (29, 30).While our patient denied the use of illicit substances, his eventual recollection of possibly ingesting contaminated food aligns with this epidemiological pattern, underscoring that accidental poisoning via environmental or food contamination is a plausible and critical route. Therefore, in any patient presenting with severe, unexplained coagulopathy—particularly with markedly elevated PT/INR and a normal or low factor VII level—a high index of suspicion for superwarfarin poisoning is warranted, even in the absence of a clear rodenticide exposure history. Prompt toxicological screening for specific LAARs is crucial for a definitive diagnosis and informs the necessity for prolonged, high-dose vitamin K1 therapy.

### 3.5 Limitations

This case report has several limitations. First, the exact source and route of brodifacoum exposure, while strongly suggested to be accidental, could not be definitively confirmed. Second, the reversal of coagulopathy was achieved using FFP and vitamin K. Although effective, the use of four-factor prothrombin complex concentrate (PCC), which is associated with more rapid and predictable correction of INR with lower transfusion volume, might have been an alternative option. The choice of FFP was guided by the institutional guidelines and formulary availability at the time. Lastly, the patient's subjective experience and quality of life impact were not systematically assessed, which could have enriched the patient-centered perspective of this report.

## 4 Conclusion and implications

The successful management of this case highlights several critical clinical imperatives. First, when encountering an aortic dissection accompanied by severe and unexplained coagulopathy, clinicians must broaden the differential diagnosis to include superwarfarin poisoning. Delay in identifying this toxicological etiology risks dissection progression and increased mortality. Second, a dual-strategy intervention is essential: rapid correction of coagulopathy with high-dose vitamin K and fresh frozen plasma, combined with stringent hemodynamic control to reduce aortic wall stress. Finally, given the exceptionally prolonged half-life of superwarfarins, long-term follow-up is mandatory to monitor aortic stability and prevent rebound coagulopathy after therapy cessation.

## Data Availability

The original contributions presented in the study are included in the article/Supplementary Material, further inquiries can be directed to the corresponding author.

## References

[1] Evangelista A Isselbacher EM Bossone E Gleason TG Eusanio MD Sechtem U. Insights from the international registry of acute aortic dissection: a 20-year experience of collaborative clinical research. Circulation. (2018) 137(17):1846–60. 10.1161/CIRCULATIONAHA.117.031264 29685932

[2] Riambau V Böckler D Brunkwall J Cao P Chiesa R Coppi G. Editor's choice—management of descending thoracic aorta diseases: clinical practice guidelines of the European society for vascular surgery (ESVS). Eur J Vasc Endovasc Surg. (2017) 53(1):4–52. 10.1016/j.ejvs.2016.06.005 28081802

[3] Karballaei Mirzahosseini H Moradpour N Yousefi Mazhin E Najafi A Mojtahedzadeh A Mojtahedzadeh M. Warfarin without therapeutic monitoring is a rodenticide, but this time it kills the patient: a warfarin toxicity case report. Arch Anesthesiol Crit Care. (2025) 11(2):244–6. 10.18502/aacc.v11i2.17972

[4] King N Tran MH. Long-acting anticoagulant rodenticide (superwarfarin) poisoning: a review of its historical development, epidemiology, and clinical management. Transfus Med Rev. (2015) 29(4):250–8. 10.1016/j.tmrv.2015.06.002 26239439

[5] Watt BE Proudfoot AT Bradberry SM Vale JA. Anticoagulant rodenticides. Toxicol Rev. (2005) 24(4):259–69. 10.2165/00139709-200524040-00005 16499407

[6] Routh CR Triplett DA Murphy MJ Felice LJ Sadowski JA Bovill EG. Superwarfarin ingestion and detection. Am J Hematol. (1991) 36(1):50–4. 10.1002/ajh.2830360111 1984683

[7] Whitlon DS Sadowski JA Suttie JW. Mechanism of coumarin action: significance of vitamin K epoxide reductase inhibition. Biochemistry. (1978) 17(8):1371–7. 10.1021/bi00601a003 646989

[8] Chong YK Mak TW. Superwarfarin (long-acting anticoagulant rodenticides) poisoning: from pathophysiology to laboratory-guided clinical management. Clin Biochem Rev. (2019) 40(4):175–85. 10.33176/AACB-19-00029 31857739 PMC6892705

[9] Mazzolai L Teixido-Tura G Lanzi S Boc V Bossone E Brodmann M. 2024 ESC guidelines for the management of peripheral arterial and aortic diseases. Eur Heart J. (2024) 45(36):3538–700. 10.1093/eurheartj/ehae179 39210722

[10] Grimm M Loewe C Gottardi R Funovics M Zimpfer D Rodler S. Novel insights into the mechanisms and treatment of intramural hematoma affecting the entire thoracic aorta. Ann Thorac Surg. (2008) 86(2):453–6. 10.1016/j.athoracsur.2008.03.078 18640315

[11] Moreno PR Purushothaman KR Fuster V Echeverri D Truszczynska H Sharma SK. Plaque neovascularization is increased in ruptured atherosclerotic lesions of human aorta: implications for plaque vulnerability. Circulation. (2004) 110(14):2032–8. 10.1161/01.CIR.0000143233.87854.23 15451780

[12] Mussa FF Horton JD Moridzadeh R Nicholson J Trimarchi S Eagle KA. Acute aortic dissection and intramural hematoma: a systematic review. JAMA. (2016) 316(7):754–63. 10.1001/jama.2016.10026 27533160

[13] Roy SB Bhatia ML Mathur VS Virmani S. Hemodynamic effects of chronic severe anemia. Circulation. (1963) 28:346–56. 10.1161/01.CIR.28.3.346 14059454

[14] Taylor CA Cheng CP Espinosa LA Tang BT Parker D Herfkens RJ. *In vivo* quantification of blood flow and wall shear stress in the human abdominal aorta during lower limb exercise. Ann Biomed Eng. (2002) 30(3):402–8. 10.1114/1.1476016 12051624

[15] Taguchi E Nishigami K Miyamoto S Sakamoto T Nakao K. Impact of shear stress and atherosclerosis on entrance-tear formation in patients with acute aortic syndromes. Heart Vessels. (2014) 29(1):78–82. 10.1007/s00380-013-0328-z 23475325 PMC3890047

[16] Ochoa JL Yarnitsky D Marchettini P Dotson R Cline M. Interactions between sympathetic vasoconstrictor outflow and C nociceptor-induced antidromic vasodilatation. Pain. (1993) 54(2):191–6. 10.1016/0304-3959(93)90208-7 8233533

[17] Chin AS Willemink MJ Kino A Hinostroza V Sailer AM Fischbein MP. Acute limited intimal tears of the thoracic aorta. J Am Coll Cardiol. (2018) 71(24):2773–85. 10.1016/j.jacc.2018.03.531 29903350 PMC6004827

[18] Xiang BT Yang Y Qiu JT Li LM Luo XL. Current status of diagnosis and treatment of aortic intramural hematoma [in Chinese]. Chin J Cardiol. (2019) 24(1):79–82.

[19] Blunt DM Impallomeni MG. Warfarin-associated thoracic aortic dissection in an elderly woman. Age Ageing. (2004) 33(2):199–201. 10.1093/ageing/afh015 14960440

[20] Bocchino PP D Filippo O Piroli F Scacciatella P Imazio M D'Ascenzo F. Anticoagulant and anti-thrombotic therapy in acute type B aortic dissection: when real-life scenarios face the shadows of the evidence-based medicine. BMC Cardiovasc Disord. (2020) 20(1):29. 10.1186/s12872-020-01342-2 31973746 PMC6977351

[21] Bhatty O Jhand AS Alkhafaji N Mooss A. Challenges in clinical decision making: a patient with type B aortic dissection presenting with pulmonary embolism. Am J Respir Crit Care Med. (2017) 195:A4179.

[22] Cañadas MV Vilacosta I Ferreirós J Bustos A Díaz-Mediavilla J Rodríguez E. Intramural aortic hematoma and anticoagulation. Rev Esp Cardiol. (2007) 60(2):201–4. Spanish. 10.1157/13099468 17338886

[23] Turgay Yıldırım Ö Çanakçı ME Aydın F Hüseyinoğlu Aydın A Akşit E. Short term follow-up of a patient with uncomplicated type B aortic dissection under dabigatran treatment. Turk Kardiyol Dern Ars. (2018) 47(5):413–6. 10.5543/tkda.2018.96892 31311911

[24] Davis DP Grossman K Kiggins DC Vilke GM Chan TC. The inadvertent administration of anticoagulants to ED patients ultimately diagnosed with thoracic aortic dissection. Am J Emerg Med. (2005) 23(4):439–42. 10.1016/j.ajem.2004.10.010 16032607

[25] Karballaei Mirzahosseini H Shahriari P Yousefi-Mazhin E Najafi A Mojtahedzadeh A Mojtahedzadeh M. Massive gastrointestinal bleeding, consequences interactions of apixaban, diltiazem and aspirin: case report. Arch Anesthesiol Crit Care. (2025) 11(2):256–8. 10.18502/aacc.v11i2.17974

[26] Ageno W Gallus AS Wittkowsky A Crowther M Hylek EM Palareti G. Oral anticoagulant therapy: antithrombotic therapy and prevention of thrombosis, 9th ed: American college of chest physicians evidence-based clinical practice guidelines. Chest. (2012) 141(2 Suppl):e44S–88. 10.1378/chest.11-2292 22315269 PMC3278051

[27] Suchard MA Schuemie MJ Krumholz HM You SC Chen RJ Pratt N. Comprehensive comparative effectiveness and safety of first-line antihypertensive drug classes: a systematic, multinational, large-scale analysis. Lancet. (2019) 394(10211):1816–26. 10.1016/S0140-6736(19)32317-7 31668726 PMC6924620

[28] Zhou XX Sun XW Ji LL Ren XD Sun YP. List of Banned and Restricted Use Pesticides in China [EB/OL]. China Pesticide Information Network, (2025-06-25). Available online at: http://www.chinapesticide.org.cn/zgnyxxw/zwb/detail/30477 (Accessed April 18, 2026).

[29] Arepally GM Ortel TL. Bad weed: synthetic cannabinoid-associated coagulopathy. Blood. (2019) 133(9):902–5. 10.1182/blood-2018-11-876839 30655273

[30] Navon L Moritz E Austin C Wahl M Aks S Layden J. The public health response to a large poisoning outbreak involving an illicit substance: synthetic cannabinoids contaminated with a long-acting anticoagulant rodenticide, Illinois, march-July, 2018. J Public Health Manag Pract. (2020) 26(6):E1–7. 10.1097/PHH.0000000000001002 30969282 PMC10926914

